# Altered Functional Performance in Patients with Fibromyalgia

**DOI:** 10.3389/fnhum.2017.00014

**Published:** 2017-01-26

**Authors:** Isis da Silva Costa, Antoni Gamundí, José G. Vivas Miranda, Lucas G. Souza França, Charles Novaes De Santana, Pedro Montoya

**Affiliations:** ^1^Research Institute of Health Sciences (IUNICS), University of the Balearic IslandsPalma de Mallorca, Spain; ^2^Department of Physics of the Earth and the Environment, Federal University of BahiaSalvador, Brazil; ^3^Motion Analysis Laboratory, Spaulding Rehabilitation Hospital, Harvard Medical SchoolBoston, MA, USA; ^4^Department of Clinical and Experimental Epilepsy, UCL Institute of Neurology, University College LondonLondon, UK; ^5^Department of Evolutionary Biology and Environmental Studies, University of ZürichZürich, Switzerland

**Keywords:** fibromyalgia, chronic pain, gait, balance, Hurst exponent, computer vision software

## Abstract

Fibromyalgia is a common chronic pain condition that exerts a considerable impact on patients' daily activities and quality of life.

**Objectives:** The main objective of the present study was to evaluate kinematic parameters of gait, functional performance, and balance in women with fibromyalgia syndrome.

**Methods:** The study included 26 female patients with fibromyalgia (49.2 ± 8.0 years) according to the criteria of the American College of Rheumatology, as well as 16 pain-free women (43.5 ± 8.5 years). Gait and balance parameters were extracted from video recordings of participants performing several motor tasks. Non-linear dynamic of body sway time series was also analyzed by computing the Hurst exponent. In addition, functional performance and clinical pain were obtained by using standardized motor tests (Berg's balance scale, 6-min walking test, timed up and go task, Romberg's balance test) and self-report questionnaires (Fibromyalgia Impact Questionnaire).

**Results:** Walking speed was significantly diminished (*p* < 0.001) in FM patients as compared to pain-free controls, probably due to significant reductions in stride length (*p* < 0.001) and cycle frequency (*p* < 0.001). Analyses of balance also revealed significant differences between fibromyalgia and pain-free controls on body sway in the medial-lateral and anterior-posterior axes (all *ps* < 0.01). Several parameters of gait and balance were significantly associated with high levels of pain, depression, stiffness, anxiety, and fatigue in fibromyalgia.

**Conclusion:** Our data revealed that both gait and balance were severely impaired in FM, and that subjective complaints associated with FM could contribute to functional disability in these patients. These findings suggest that optimal rehabilitation and fall prevention in fibromyalgia require a comprehensive assessment of both psychological responses to pain and physical impairments during postural control and gait.

## Introduction

Fibromyalgia (FM) is a chronic syndrome characterized by widespread pain sensitivity and fatigue, as well as by cognitive and affective symptoms (Wolfe et al., 2010). Fibromyalgia also exerts a considerable impact on daily activities and quality of life. In particular, it has been frequently shown that fatigue in fibromyalgia may be severe enough to reduce physical activities and lead to a sedentary lifestyle by reducing physical abilities and increasing risk for disabilities (Bennett et al., 2007; Jones et al., 2008). FM patients often reported functional limitations that were quite similar to those reported by persons with osteoarthritis or rheumatoid arthritis (Hawley and Wolfe, 1991). Furthermore, it has been shown that loss of function could be strongly associated with work disability in these patients (White et al., 1999; Wolfe and Michaud, 2004).

Previous research has also revealed that FM patients may also display deficits in balance or postural stability (Bennett et al., 2007; Jones et al., 2009; Russek and Fulk, 2009), a complex task that involves rapid and dynamic integration of multiple sensory, motor, and cognitive inputs to execute appropriate neuromuscular activity (Horak, 2006; Sousa et al., 2012). Impaired balance has been reported as one of the top ten debilitating symptoms in fibromyalgia with prevalence rates around 45% (Bennett et al., 2007). Moreover, frequency of falls seems to be higher in FM patients (34.4%) (Russek and Fulk, 2009) than in persons aged 65 years and older (25–35%) (Sattin, 1992) and patients with rheumatoid arthritis (Hawley and Wolfe, 1991). Nevertheless, balance and activity level in fibromyalgia have been mostly assessed by using retrospective self-reports (Mannerkorpi et al., 1994; Russek and Fulk, 2009), which are strongly influenced by patients' beliefs about their own physical functioning and pain (Verbunt et al., 2003). In the last decades, different types of recording devices have been developed to monitor and to assess balance and physical activity over long periods of times, providing valid information about subjects' daily activities. Thus, it has been demonstrated that accelerometry-based ambulatory monitoring systems provide more objective measurements of variability in physical activities and pain over several days than self-reports (Verbunt et al., 2009). Biomechanical analysis of gait also constitutes a useful tool for the assessment of motor function, functional capacity and muscle fatigue (Bendtsen et al., 1997; Pierrynowski et al., 2005; Sousa et al., 2012). Previous studies have observed that fibromyalgia women display a reduced walking speed, which could be a consequence of decreases in stride length and cycle frequency, as well as bradykinesia (Auvinet et al., 2006; Heredia Jiménez et al., 2009). Furthermore, it has been suggested that gait at normal speed in these patients may be preferentially achieved by using their hip flexors instead of their ankle plantar flexors, thus increasing metabolic demands and fatigue in comparison to pain-free controls (Pierrynowski et al., 2005). Despite the evidence of altered gait and balance parameters in FM, little is known about how these abnormalities could be linked to clinical variables such as pain, fatigue, stiffness, or depression.

The aim of the present study was to analyze gait and balance parameters in fibromyalgia and to examine the possible relationship between subjective and objective measures of motor function with subjective complaints. In particular, we hypothesized that FM patients would display significant gait and balance deficits as compared with pain-free controls, and that these motor disturbances would be correlated with increased patients' ratings of pain, fatigue, morning tiredness, stiffness, and physical impairment (as measured by the FIQ questionnaire). Furthermore, considering that activity and balance fluctuations have well defined fractal properties in a wide range of time scales, we also aimed to apply nonlinear analyses to evaluate the dynamic of these balance fluctuations in pain-free controls and FM patients. This nonlinear approach allows an evaluation of the autocorrelation in successive displacements, giving us information about possible disturbances in motor control mechanism to correct balance.

## Materials and methods

### Participants

Twenty-six women diagnosed with fibromyalgia and 16 pain-free women with comparable age and sociodemographic characteristics were recruited from different health centers and patients' associations in Majorca (Spain). The average duration of FM diagnosis was 10.8 ± 7.7 years Patients were included in the study if they fulfilled the 1990 classification criteria of the American College of Rheumatology for fibromyalgia. Participants were excluded from the study if they reported any other musculoskeletal rather than fibromyalgia, or any neurological disorder. Regarding medication intake, most FM patients were taking analgesics, relaxants, or NSAIDs (*n* = 18), followed by antidepressants (*n* = 16), and anxiolytics (*n* = 9). For medical and ethical reasons, medication was not discontinued during the study. At the time of recruitment, all participants were verbally informed about the details of the study and provided written consent. The study was approved by the Ethics Committee of the Balearic Islands (Spain) (reference IB-1284/09).

### Self-report questionnaire

FM patients completed the *Fibromyalgia Impact Questionnaire (FIQ)* (Burckhardt et al., 1991). The FIQ is a standardized instrument designed to quantify the overall impact of fibromyalgia. Subscales from the 1991 version include 11 physical function items (4-point Likert scale ranging from “always” to “never”), feel good (number of days of the past week), missed work (number of work days in the past week), and 7 symptom-based items (ability to do job, pain, fatigue, rested, stiffness, anxiety, and depression) (100-mm anchored visual analog scale). Test-retest correlations using Pearson's r ranged from 0.56 (pain) to 0.95 for physical function scale. This questionnaire has shown excellent responsiveness to change in clinical studies and a good correlation with other similar questionnaires such as the SF-36 (Bennett, 2005).

### Motor function tasks

Gait and balance parameters were obtained in FM patients and pain-free controls by using the following functional tasks:

- *Berg Balance Scale* (Berg et al., 1991): This scale is a performance-based assessment tool developed to measure balance during functional activities such as reaching, bending, transferring, and standing. The test is often used for patients who exhibit a decline in function, self-report a loss of balance, or have unexplained falls (Berg et al., 1991). The Berg Balance Scale consists of 14 functional tasks (e.g., sitting unsupported, change of sitting to standing position, and vice-versa, standing with both feet together, standing on one leg, turning 360 degrees) with scores ranging from 0 (unable to perform) to 4 (normal performance). Total scores range from 0 (severely impaired balance) to 56 (excellent balance). Scores below 46 are good predictors for the occurrence of multiple falls (Dibble et al., 2008).
- *Six-minute walking test (6MWT)*: The 6MWT is a functional walking test in which subjects are instructed to walk for 6 min as quickly as possible. This test has been used to assess individuals with mobility deficits (Kosak and Smith, 2005) and FM patients (King et al., 1999; Pankoff et al., 2000a; Latorre-Román et al., 2014). The 6MWT is considered a good indicator of exercise tolerance and aerobic capacity, since it causes a physiological stress without demanding maximum aerobic capacity (Pankoff et al., 2000a). Ratings of perceived exertion were obtained after the 6MWT by using the Borg Effort Scale (Borg, 1982), a 15-point scale ranging from 4 (complete lack of effort) to 20 (maximum effort or exhaustion).
- *Timed up and go task (TUG)*: This task is a standardized test for assessment of functional mobility. The task is performed by using an ordinary armchair (45 cm in height) and a stopwatch. Subjects are seated with their back against the chair and instructed to stand up, walk three meters, turn around, walk back to the chair, and sit down at an ordinary comfortable speed (Shumway-Cook et al., 2000). The stopwatch is started on the word “*Go*” and stopped as the subject sit down. The TUG time is measured in seconds and normal TUG time ranges from 5.4 to 40.8 s (mean = 15 s, *SD* = 6.5) (Khasnis and Gokula, 2003). TUG time appears to be correlated with gait speed, balance, functional level, and the ability to go out (Newton, 1997). After the TUG, overall subjective perception of physical effort was measured by using the Borg Effort Scale (Borg, 1982).
- *Modified version of the Romberg's balance test*: The Romberg's test is an objective measure of patient's standing balance (Khasnis and Gokula, 2003). The original test requires that participants remain in orthostatic position with feet together and eyes closed. In the present study, we modified the procedure by asking the participants to keep the erect position with eyes closed during 1 min. In addition, they were allowed to keep the orthostatic position with feet in parallel and separated and arms extended along the body to avoid that participants fell when they closed their eyes during data collection. The test is based on the fact that maintaining balance while standing with closed eyes should rely on intact sensorimotor integration and motor pathways. The test was repeated twice and motion on the frontal and sagittal planes was captured by using a digital video camera at 30 frames per second (Casio Exilim EX-FS10). For motion detection analysis, a plumb line hanging on the ceiling at a distance of 3 meters was used as reference. Participants were also asked to wear a cap with sticks positioned in the vertical and horizontal planes. For the analysis of body sway in the medial-lateral direction, sticks were aligned with the anatomical position of the glabella of the frontal bone. For the analysis of body sway in the anterior-posterior direction, sticks were aligned with the anatomical position of the pinna (tragus). Unfortunately, we were not able to analyze the Romberg's test videos of eleven FM patients and two pain-free subjects due to poor recording quality.
- *Gait task*: Subjects were instructed to walk on a 3 meters carpet at their normal walking step, without shoes and with flexed arms positioned on the abdomen. Optical markers were attached at the following body positions: anterior superior iliac spine, posterior superior iliac spine, area between the lateral condyle of the femur and the fibular head, bottom of the patella, lateral, and inner malleolus, heel (between the first and second metatarsal), and on the tip of the hallux. Subject's motion was digitally recorded with a video camera at 210 frames per second (Casio Exilim EX-FS10). The camera was positioned at a distance of 3 meters from the carpet to visualize changes in position, velocity, and acceleration of anatomical points along the x-axis. Gait velocity (cm/second), walking duration (seconds), cadence (number of steps/minute), percentage of time in the two phases of the gait cycle (stance and swing phase), and percentage of time with single and double support were computed.

### Data reduction and pre-processing

Three groups of variables were analyzed in the present study:

- Raw scores obtained from self-report questionnaire (FIQ).
- Performance scores on standardized motor function tasks (TUG, 6MWT, Berg Balance Scale, Borg Effort Scale).
- Kinematic parameters extracted from video recordings: gait velocity (cm/sec), gait duration (sec), cadence (steps/min), stride, and step lengths (cm), percentage of time in the stance/swing phase, and body sway variability in the anterior-posterior and medial-lateral planes (cm). Open-source software for computer vision analysis of human movement (CvMob; Peña et al., 2013; Gea et al., 2014; Quixadá et al., 2016) was used to extract those variables. This software has a high degree of accuracy for calculating body position and movement in the X and Y coordinates recorded by conventional cameras (Peña et al., 2013).

The non-linear dynamic of time series obtained during the balance test was also assessed by computing long term correlations and the *Hurst exponent* (Feder, 1988). This exponent usually ranges between 0 and 1, and describes the tendency of a time series either to cluster in one direction or to regress strongly to the mean. Thus, it has been assumed that Hurst exponents between 0.5 and 1 would be characteristic of time series with long-term positive autocorrelation (high values will be followed by high values a long time in the future), whereas exponents between 0 and 0.5 would suggest long-term switches between high and low values in adjacent pairs of data. By contrast, Hurst exponents would be around 0.5 if time series describe a pure random oscillation (e.g., Brownian noise or accumulated white noise). Moreover, it has been assumed that exponents lesser than 0.5 would reflect a non-persistent pattern, whereas exponents greater than 0.5 would rather reflect a persistent pattern within the time series (Feder, 1988).

The Hurst exponent was obtained in two steps. First, the deviation of the time series relative to their mean values was computed in a sliding window of size *n* by using the Root Mean Square (RMS) method (Russ, 1994). The RMS uses the scaling function $\left(\bar{W}\left(n\right)\right)$ defined as follows:

(1) $$\begin{array}{l} \bar{W}\left(n\right)=\;\frac{1}{N-n}\overset{N-n}{\underset{u=1}{\sum }}{\left\{\frac{1}{n}\overset{n}{\underset{i=1}{\sum }}{\left[Z\left(x_{u+i}\right)-{\bar{Z}}_{n}\right]}^{\text{2}}\right\}}^{1/2} \end{array}$$

with the factor *N* representing the total number of measurements and ${\bar{Z}}_{n}\;$ the average value within each scale. Second, the values for $\;\bar{W}\left(n\right)$ were evaluated for different scales *n*. The Hurst exponent was obtained by fitting a power-law curve (fractal Brownian motion model) to the scaling function (Feder, 1988; Russ, 1994) as follows:

(2) $$\begin{array}{l} \bar{W}\left(n\right)\sim n^{H} \end{array}$$

### Statistical analyses

The null hypothesis that data were sampled from a normally distributed population was examined by using Shapiro-Wilk tests, and differences between patients and pain-free controls were analyzed by using parametric Student *t*-tests for independent samples, or non-parametric two-sample Kolmogorov-Smirnov tests. Pearson correlations were also used to analyze the relationship between kinematic parameters and clinical symptoms in fibromyalgia. A *p*-value of 0.05 was used for statistical significance. The effect sizes *d* were interpreted using the classification of Cohen (1988): 0.2 ≤ *d* < 0.5 small effect, 0.5 ≤ *d* < 0.8 moderate effect, *d* ≥ 0.8 large effect. Means and standard deviations are displayed in the tables. If appropriate, data are reported as mean difference and 95% confidence interval (95% CI).

## Results

Fibromyalgia patients and pain-free controls were comparable on age (49.2 years ± 8.0 vs. 43.5 years ± 8.4, respectively), weight (68.6 kg ± 10.9 vs. 64.2 kg ± 10.9), height (161.1 cm ± 6.4 vs. 163.3 cm ± 7.0), and body-mass index (26.5 kg/m^2^ ± 4.2 vs. 25.5 kg/m^2^ ± 4.1) (all *ps* > 0.05). Mean and standard deviation of FIQ scores in fibromyalgia patients are displayed in Table 1.

**Table 1:** **Mean and standard deviations of FIQ scores in FM patients**.

	**Fibromyalgia patients *N* = 26**
**FIBROMYALGIA IMPACT QUESTIONNAIRE (FIQ*****)***	
Physical impairment (0–3)	1.6 ± 0.7
Feel good (0–7)	5.1 ± 1.7
Missed work (0–7)	2.1 ± 2.2
Do job (10 cm VAS)	8.3 ± 2.4
Pain (10 cm VAS)	8.2 ± 1.9
Fatigue (10 cm VAS)	8.9 ± 2.0
Rested (10 cm VAS)	8.1 ± 3.1
Stiffness (10 cm VAS)	7.6 ± 2.9
Anxiety (10 cm VAS)	7.6 ± 2.9
Depression (10 cm VAS)	6.8 ± 3.4
Total FIQ score (0–100)	71.1 ± 16.1

Table 2 displays mean and standard deviation of gait parameters in fibromyalgia and pain-free controls during performance on several motor tasks. FM patients walked less distance in 6 min (6MWT) [*t*_(29)_ = −8.3, *p* < 0.001], and took more time to stand-up and to walk a distance of 3 meters (TUG) as compared with pain-free controls [*t*_(40)_ = 6.7, *p* < 0.001]. Moreover, ratings on self-perceived effort (Borg Effort scale) after performance on 6MWT (K-S = 1.5, *p* < 0.05) and TUG tests (K-S = 3.02, *p* < 0.001) were significantly higher in fibromyalgia than in pain-free controls. Finally, FM patients reported increased risk of falls (measured by the Berg Balance Scale) in comparison with pain-free controls (K-S = 2.9, *p* < 0.001). The effect sizes were medium-to-large for all group comparisons.

**Table 2:** **Mean and standard deviations of gait parameters during motor performance in fibromyalgia patients and pain-free controls**.

	**Fibromyalgia patients *N* = 26**	**Pain-free controls *N* = 16**	**Cohen's d**	**Effect-size r**
**STANDARDIZED MOTOR FUNCTION TESTS**				
Berg scale for risk of falls (0–56)	44.7 ± 5.6	55.4 ± 0.6	−2.68	0.80
TUG (sec)	17.0 ± 5.2	8.2 ± 1.0	2.35	0.76
Perceived effort after TUG (4–20)	12.3 ± 2.3	4.3 ± 0.5	4.80	0.92
6MWT (m)	170.9 ± 46.9	330.1 ± 58.3	−3.00	0.83
Perceived effort after 6MWT (4–20)	14.1 ± 3.6	9.2 ± 3.1	1.45	0.59
**GAIT PARAMETERS**				
Gait velocity (cm/sec)	67.3 ± 17.3	112.0 ± 12.3	−2.97	0.82
Gait duration (sec)	4.8 ± 1.4	2.7 ± 0.3	2.07	0.72
Cadence (steps/min)	96.6 ± 19.4	115.5 ± 11.6	−1.18	0.51
Stride Length (cm)	69.3 ± 14.3	99.2 ± 12.9	−2.19	0.74
Step Length (cm)	58.6 ± 10.5	79.7 ± 8.6	−2.20	0.74
Single support (%)	57.3 ± 7.0	66.1 ± 4.2	−1.52	0.60
Swing phase (%)	29.3 ± 3.1	33.7 ± 3.0	−1.44	0.58

Analyses of kinematic parameters further indicated that FM patients had significant deficits in gait and balance. Again, the effect sizes were medium-to-large for all group comparisons. FM patients displayed significant reductions in gait velocity [*t*_(31)_ = −8.3, *p* < 0.001], cadence (steps/minute) [*t*_(31)_ = −6.2, *p* < 0.001], stride length [*t*_(31)_ = −5.1, *p* < 0.001), step length [*t*_(31)_ = −4.9, *p* < 0.001], and percentage of single support [*t*_(31)_ = −4.3, *p* < 0.001] and swing phase [*t*_(31)_ = 4.2, *p* < 0.001], as well significant increased gait duration [*t*_(31)_ = 5.7, *p* < 0.001] in comparison with pain-free participants. Same effects were also yielded when values were referenced to each subject's legs (distance between the greater Trochanter and the lateral Malleolus) (Table 2). Moreover, FM patients displayed greater body sway in the anterior-posterior [*t*_(27)_ = 4.6, *p* < 0.001] and medial-lateral directions [*t*_(27)_ = 5.8, *p* < 0.001] than pain-free controls.

**Figure 1:**
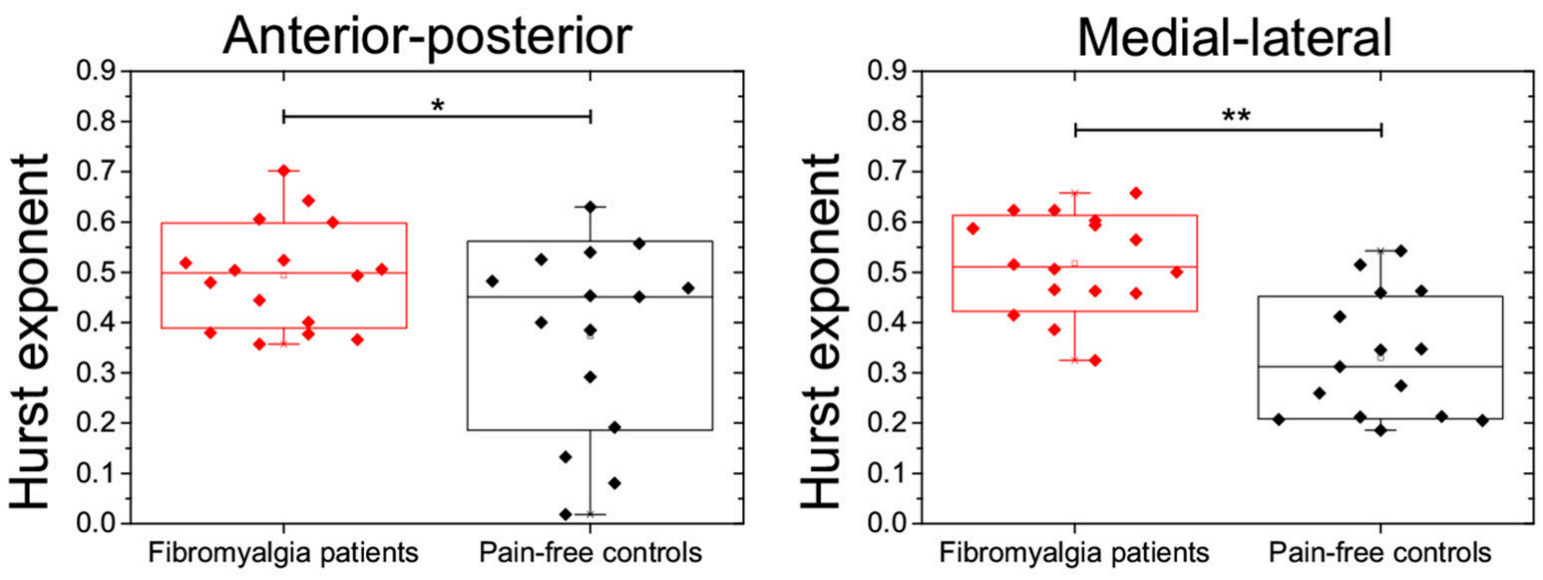
**Boxplots showing the distribution of Hurst exponents during body sways in the antero-posterior (left) and medial-lateral axes (right) in FM patients and pain-free controls**. Asterisks indicate statistical differences at ^*^p < 0.05 or ^**^p < 0.01.

The non-linear analysis of balance time series also revealed significant group differences on Hurst exponents of anterior-posterior [*t*_(27)_ = 2.3, *p* < 0.05] and medial-lateral axes [*t*_(27)_ = 5.1, *p* < 0.001]. In both cases, the *H* exponents were close to 0.5 in fibromyalgia patients and around 0.3 in pain-free controls (Figure 1 and Table 3). The effect sizes were medium-to-large for all group comparisons.

**Table 3:** **Mean and standard deviations of balance parameters in the anterior-posterior and the medial-lateral axes during motor performance in fibromyalgia patients and pain-free controls**.

	**Fibromyalgia patients *N* = 15**	**Pain-free controls *N* = 15**	**Cohen's d**	**Effect-size r**
**ANTERIOR-POSTERIOR AXIS**				
Body sway (cm)	2.31 ± 0.99	1.07 ± 0.11	1.76	0.66
Hurst exponent	0.50 ± 0.10	0.37 ± 0.19	0.85	0.39
**MEDIAL-LATERAL AXIS**				
Body sway (cm)	2.55 ± 0.92	1.08 ± 0.09	2.25	0.75
Hurst exponent	0.52 ± 0.09	0.33 ± 0.12	1.79	0.67

In order to further assess if altered motor function was related to clinical symptoms in fibromyalgia, Pearson correlations were computed between motor performance scores and ratings on the different FIQ scales. Results indicated that high ratings on pain intensity were significantly associated with enhanced risk of falls (Berg Balance Scale, *r* = −0.52 and *p* < 0.01), increased time to perform the TUG test (*r* = 0.44, *p* < 0.05), reduced distance to walk in 6 min in 6MWT test (*r* = −0.53, *p* < 0.05), gait velocity (*r* = −0.56, *p* < 0.05), cadence (*r* = −0.50, *p* < 0.05) and stride lengths (*r* = −0.49, *p* < 0.05), as well as increased gait duration (*r* = 0.54, *p* < 0.05) and body sways in the medial-lateral axis (*r* = 0.68, *p* < 0.05). High ratings on fatigue and stiffness were also associated with reduced percentage of single support (*r* = −0.52, *p* < 0.05 and *r* = −0.48, *p* < 0.05, respectively). In addition, high ratings on stiffness were related to enhanced perceived effort after completion of the 6MWT test (*r* = 0.60, *p* < 0.05), and reduced stride (*r* = −0.56, *p* < 0.01) and step lengths during the gait cycle (*r* = −0.54, *p* < 0.05). High ratings on the physical function scale were significantly associated with high risk of falls (Berg Balance Scale, *r* = −0.39 and *p* < 0.01) and increased perceived effort after completion of the TUG (*r* = 0.56, *p* < 0.01), as well as with reduced distance walked in 6 min during performance of 6MWT test (*r* = −0.50, *p* < 0.05). The number of missed days of work were significantly associated with high risk of falls (Berg Balance Scale, *r* = −0.40 and *p* < 0.05), enhanced perceived effort after completion of the 6MWT test (*r* = 0.60, *p* < 0.05) and reduced distance to walk in 6 min in 6MWT test (*r* = −0.50, *p* < 0.05). Low ratings on ability to do job were significantly associated with high risk of falls (Berg Balance Scale, *r* = −0.47 and *p* < 0.05) and increased body sways in the medial-lateral axis (*r* = 0.53, *p* < 0.05). High ratings on the rested scale were significantly associated with reduced distance walked in 6 min during performance of 6MWT test (*r* = −0.57, *p* < 0.05). Finally, ratings on depression were correlated with risk of falls (Berg Balance Scale, *r* = −0.46 and *p* < 0.01), increased time to perform the TUG test (*r* = 0.49, *p* < 0.05), enhanced perceived effort after completion of TUG (*r* = 0.49, *p* < 0.05), and reduced stride (*r* = −0.53, *p* < 0.05) and step lengths (*r* = −0.49, *p* < 0.05). High ratings on anxiety were significantly associated with high risk of falls (Berg Balance Scale, *r* = −0.55 and *p* < 0.01) and increased body sways in the medial-lateral axis (*r* = 0.54, *p* < 0.05).

## Discussion

We analyzed kinematic parameters of gait and balance, as well as subjective complaints (ratings of perceived exertion, pain, fatigue, stiffness, depression, anxiety) during performance on several motor and balance tasks in fibromyalgia patients and age-matched pain-free controls. Our results indicated that both gait and balance were severely impaired in FM, and that several parameters of motor performance were linked to clinical symptoms associated with FM.

Gait parameters such as speed, cadence, stride and step lengths, percentage of stance, and swing phases, and support base were significantly impaired in FM patients. These findings are in accordance with previous studies showing that FM patients displayed slower cadence during gait compared to pain-free controls (Pankoff et al., 2000b; Auvinet et al., 2006; Heredia Jiménez et al., 2009). Furthermore, it has been reported that FM women spent more time in double than in single support, as well as reduced muscle endurance and both isometric and isokinetic strength in knee joint flexion and extension (Valkeinen et al., 2008; Heredia Jiménez et al., 2009; Cherry et al., 2012). In addition, it has been suggested that generalized pain and overweight could inhibit the single support of body and increase the time of double support in FM (Heredia Jiménez et al., 2009; Cherry et al., 2012). This is of special relevance because the preferential use of hip flexors in comparison to plantar flexors of the ankle in FM patients would also indicate an altered mechanism for maintaining balance during gait (Winter, 1995; Pierrynowski et al., 2005; Valkeinen et al., 2008). Previous studies have also suggested that factors such as level of physical activity, bradykinesia and overweight, together with fatigue and pain could be also responsible for relevant alterations in muscle recruitment patterns during gait in FM (Pierrynowski et al., 2005; Auvinet et al., 2006; Heredia Jiménez et al., 2009). In this sense, our findings were consistent with previous studies showing that patients with chronic pain displayed a reduced level of activity during the morning and the evening compared to pain-free controls (Weering et al., 2009). It was also noteworthy that observed alterations of gait parameters in FM (for instance, a reduction of more than 30% in gait velocity and stride length compared to age-matched healthy individuals) were similar or even greater than those previously reported during aging (for instance, a reduction of 20% in older as compared to young individuals) (Elble et al., 1991; Li et al., 2011). Thus, it seems plausible that an altered pattern of gait could also contribute to the characteristic reductions of daily functioning in FM.

The analysis of body sway during performance on the modified version of the Romberg's balance test further supports the notion that FM may affect some subsystems responsible for postural control and balance. Body sways on the anterior-posterior and medial-lateral axes were significantly greater in FM patients than in pain-free controls. Furthermore, non-linear analyses of body sway time series showed that Hurst exponent values were significantly lower in pain-free controls (values ranging between 0.3 and 0.4) than in FM patients (values around 0.5). These findings were in agreement with previous data observed in healthy individuals (Duarte and Zatsiorsky, 2000) and patients with reduced mobility (Burgunder, 1998; Stylianou et al., 2011). Basically, Hurst exponents below 0.5 would indicate that shifts of the time-series in one direction are followed by shifts in the opposite direction, revealing an anti-persistent trend of body sway to maintain a stable body position along the time. By contrast, Hurst exponents close to 0.5 in FM patients would indicate that time-series were characterized by an uncorrelated pattern of body sway leading to a more unstable balance. This uncorrelated or random behavior may suggest the existence of relevant disturbances in the motor control system which could lead to an increased risk of falls in these patients. Our findings from the Timed Up and Go (TUG) task are also in agreement with this interpretation. We observed that FM patients took significantly more time to complete the task (around 17 s) than pain-free controls (8 s). These values were similar to those obtained in a previous study (Shumway-Cook et al., 2000) showing that older people performing the TUG in more than 13.5 s were more likely to have suffered a fall in the previous 6 months. The analyses of balance during functional activities (reaching, bending, transferring, and standing) further indicated that FM patients displayed higher risk of falls than pain-free controls. In this sense, it has been already reported that balance deficits could be considered as one of the top 10 most debilitating symptoms in FM (Bennett et al., 2007). Moreover, the observed values for risk of falls in the present study were similar to those previously reported in the elderly (Berg et al., 1991; Panton et al., 2006) and in Parkinson patients (Dibble et al., 2008; Fernandes et al., 2015). Taking into account that around 30% of people over 65 may fall at least once a year (Mannerkorpi et al., 1994; Sylliaas et al., 2009), one may speculate that risk of falls in FM patients could represent an important limitation in their elderly life.

Although the influence of psychological factors on motor disturbances observable in chronic pain is still unclear, a common assumption is that pain catastrophizing, hypervigilance, fear of pain, and subsequent avoidance of activities that are known to exacerbate pain (fear-avoidance model) might contribute to reduce physical activity and to alter gait and balance parameters (such as muscle weakness, slower walking, shorter step length, shorter stride time, or higher trunk muscle activity) in chronic back pain (Vlaeyen and Linton, 2000; Leeuw et al., 2007). In line with these previous findings, our data show that FM patients exhibited objective alterations in gait and balance, which were associated with frequent complaints such as pain, stiffness, fatigue, depression, and anxiety. Nevertheless, our findings seem to suggest that gait and balance deficits could be related to different subjective FM complaints. Thus, for instance, reduced stride length and increased time taken to perform the TUG task were linked to high pain intensity, depression and stiffness, whereas increased body sway in the medial-lateral axis was positively associated with pain intensity and anxiety. In addition, other gait parameters such as gait velocity, gait duration or cadence were only associated with pain intensity, and body sways in the anterior-posterior axis or Hurst exponents of body sways in both axes were even not correlated with pain-related complaints in FM patients. These differences may reflect a differential effect of depression and anxiety on gait and balance and warrant further investigation in FM patients. Moreover, analyses of gait and balance may provide additional information for the identification of subgroups among fibromyalgia patients based on psychosocial and cognitive characteristics (Auvinet et al., 2006). Therefore, multidisciplinary interventions for fibromyalgia should include a focus on correcting functional deficits and instilling greater self-confidence in patients to engage in physical exercise to improve functional outcomes.

The present study has some limitations that should be taken into account for the interpretation of the results. Two-thirds of our FM patients were currently taking analgesic and antidepressant medication during data collection and, therefore, the possible side effects of these drugs on balance and gait cannot be completely discarded. In this sense, a recent study has shown that antidepressant use was one of the possible mediators for the association between obesity and risk of falls in community living older persons (Mitchell et al., 2015). It remains, however, unclear if similar effects could be observable in middle-age FM patients. Moreover, although our sample of FM patients displayed greater body-mass index than age-matched pain-free controls, they could not be considered as obese. Although prevalence of FM in men is significantly lower than in women, future studies should include representative samples of men, as well as medication-free and older participants to examine the mediator role of all these variables on gait and balance. Finally, it should be borne in mind that fatigue was assessed as a subjective symptom from the FIQ questionnaire. Further research is necessary to analyze if more objective and reliable measures of fatigue are also correlated with gait deficits in FM.

In conclusion, our results point toward significant impairments in balance in FM patients as compared with pain-free controls, as assessed by self-reports, standardized motor function tests and kinematic parameters extracted from participants' video recordings. We found that pain intensity, stiffness, fatigue, depression and anxiety were the most relevant factors in explaining some gait and balance deficits in FM. We have also found that FM patients displayed an abnormal pattern of body sways during a balance task, which could be associated with changes in the motor control system and explain a higher risk of falls. All these findings highlight the relevant role of postural control and balance for daily activity functioning in FM. Thus, specific activities directed toward the modification of these altered gait and balance patterns may be included in regular physical intervention programs for FM. This represents a relevant contribution considering that most of previous research on functional disability in FM was based on retrospective reports or on self-report measures rather than on objective measures of gait and balance.

## Author contributions

IdSC designed the study, conducted the experiments and wrote the first version of the manuscript. All the authors contributed substantially to data analysis, interpretation of the results and critical revisions of the manuscript. The final version was approved by all authors. All authors agreed to be accountable for all aspects of the work in ensuring that questions related to the accuracy or integrity of any part of the work are appropriately investigated and resolved.

### Conflict of interest statement

The authors declare that the research was conducted in the absence of any commercial or financial relationships that could be construed as a potential conflict of interest.
